# Hypomagnesemia-Induced Cerebellar Syndrome—A Distinct Disease Entity? Case Report and Literature Review

**DOI:** 10.3389/fneur.2020.00968

**Published:** 2020-09-08

**Authors:** Christian P. Kamm, Thomas Nyffeler, Christoph Henzen, Stefan Fischli

**Affiliations:** ^1^Neurology and Neurorehabilitation Center, Luzerner Kantonsspital, Lucerne, Switzerland; ^2^Department of Neurology, Inselspital, Bern University Hospital and University of Bern, Bern, Switzerland; ^3^Division of Endocrinology, Diabetes and Clinical Nutrition, Luzerner Kantonsspital, Lucerne, Switzerland

**Keywords:** cerebellar syndrome, hypomagnesemia, oedema, MRI, literature review, recurrent disease, ataxia

## Abstract

Clinical consequences of hypomagnesemia are manifold and include various neurological syndromes up to life-threatening conditions. Despite its importance, magnesium is generally not routinely determined leading to an under-recognition of hypomagnesemia-related disorders. In the past years, there are growing numbers of reports of hypomagnesemia-induced cerebellar syndromes (HiCS) with corresponding cerebellar edema, which might be a distinct disease entity. To provide further insights into HiCS, we describe a patient with HiCS and performed a literature review on cerebellar syndromes due to severe hypomagnesemia with regard to the clinical, MRI, and laboratory findings. We identified 17 cases, so including our case, 18 cases contribute to this review. Summarized, HiCS seems to be a distinct disease entity because of the remarkable similarities of clinical, MRI, and laboratory features. It should be diagnosed and treated early to avoid recurrent disease courses, residual symptoms, and potentially life-threatening conditions such as seizures. Physicians must be alert to HiCS as magnesium is usually not part of the routine electrolyte panel.

## Introduction

Magnesium (Mg) plays an important role in many biochemical and physiological processes particularly in the brain, heart, and skeletal muscle (1). Being the second most abundant intracellular cation after potassium, it is involved in over 600 enzymatic reactions including energy metabolism, protein synthesis, stabilizing vascular endothelium, and regulating neurotransmitter function (1, 2). As an essential ion to the human body, Mg hemostasis is strictly regulated by the uptake in the small intestine and the excretion in the kidney.

Clinical consequences of hypomagnesemia are manifold and include electrolyte abnormalities (hypokalemia, hypocalcemia), arrhythmias (ventricular arrhythmias, torsade de pointes, supraventricular tachycardia), and various neurological syndromes such as carpopedal spasm, muscle cramps, muscle fasciculations, myopathy, vertigo, nystagmus, choreoathetosis, hemiparesis, depression, delirium, and seizures (1, 2). Despite its importance, serum Mg values are generally not routinely determined leading to an under-recognition of hypomagnesemia-related disorders (2).

In recent years, there is a growing number of reports of hypomagnesemia-induced cerebellar syndromes with corresponding cerebellar edema seen on MRI which might be a distinct disease entity (3–17).

We describe a 74-year-old man with recurrent cerebellar syndrome due to hypomagnesemia with regard to the clinical course, laboratory analysis, and MRI characteristics. Also, we performed a literature review on cerebellar syndromes due to severe hypomagnesemia to provide insights into this pathologic entity.

## Case Description

### First Episode 2015

In July 2015, a 71-year-old man was admitted to the hospital due to vertigo, walking difficulties, dysarthria, nausea, and vomiting for 3 weeks. A brain MRI 2 weeks before admission was normal. On admission, the patient was fully orientated with a normal afebrile general condition (blood pressure 120/81 mmHg, pulse 86/min). The neurological examination revealed severe dysarthria and gait ataxia (walking only with support).

Pre-existing conditions were type 2 diabetes mellitus, hypertension, hyperlipidemia, intermittent hypokalemia, vitamin B_12_ deficiency, monoclonal gammopathy (MGUS), and small-fiber polyneuropathy with secondary restless legs syndrome (Table 1). Consequently, the patient was on the following medications: esomeprazole, rosuvastatin, acetylsalicylic acid, pramipexole, lorazepam, solifenacin, sitagliptin/metformin, potassium chloride, and metoclopramid.

**Table 1:** Clinical summery of reported cases.

**References**	**Age (y)**	**Sex (m/f)**	**Symptoms before admission**	**Findings at admission**	**Comorbidities**	**Etiology**	**Therapy**	**Clinical course**		
								**Improvement**	**Recurrence**	**Residual symptoms**
Case report	71	m	Subacute vertigo, dysarthria, nausea, impaired walking	Dysarthria, gait ataxia	HT, T2DM, PNP, RLS, MGUS dyslipidemia	PPI	Mg p.o. PPI stop	y	y	Vertigo, nausea
(3)	57	m	Subacute dysarthria, gait ataxia	Dysarthria, nystagmus, ataxia, cognitive impairment, HT	Alcohol abuse, pancreatitis, liver steatosis, seizure	Alcohol abuse + withdrawal	Mg i.v.	y	n	Dysarthria, nystagmus, gait ataxia
(4)	59	m	Sudden-onset vertigo, nausea, tinnitus, falls	Intermittent DBN, ataxia, somnolence, seizures, arrhythmia	HT, T2DM, ulcerative colitis, ileostomy	Intestinal malabsorption	Mg + Ca i.v.	y	n	
(5)	49	m	Subacute nausea/vomiting, diplopia, headache	Nystagmus, tachycardia, somnolence, ataxia, hydrocephalus, HT	Alcohol abuse, HT, hyperlipidemia, GERD	PPI, alcohol abuse	Mg i.v.	y	y	Mild ataxia left hand
(5)	60	m	Vertigo, oscillopsia, vomiting, unsteadiness	DBN, limb ataxia, gait ataxia	Alcohol abuse, T2DM, HT, stroke	PPI	Mg i.v. PPI stop	y	n	Broad-based gait
(5)	80	w	Seizure, dizziness, vomiting	DBN, ataxia	HT, Parkinson's disease, systemic sclerosis, GERD	PPI	Mg	y	n	Dizziness, nystagmus
(6)	38	m	Dizziness, gait ataxia, oscillopsia	DBN, limb/gait ataxia dysdiadokinesia	HT	PPI	Mg i.v. PPI stop	y	y	
(7)	68	w	Vertigo, visual blurring, nausea/vomiting	Periodic DBN, seizures dysmetria, somnolence	Scleroderma, diarrhea, vomiting, weight loss	Small intestine resection	Mg i.v., Ca, Po	y	n	No
(8)	43	m	Dysarthria, ataxia, nausea, headache, mental disorder	Confusion, delirium, dysarthria, hyperreflexia, ataxia	T2DM, smoking	PPI	Mg + Ca i.v. PPI stop	y	n	Cognition, ataxia
(9)	72	m	Progressive dysarthria, ataxia, headache	Nystagmus dysarthria, dysphagia, dysmetria, gait ataxia	Sigmoid carcinoma, metastases, weight loss, colon resection	PPI, short bowel syndrome	Mg	y	n	No
(10)	73	w	Nausea, dizziness, lethargy, confusion	Cerebellar syndrome, seizure, arrhythmia	T2DM, weight loss, hyperlipidemia, HT	PPI	Mg i.v.		y	
(11)	68	w	Vertigo, nausea, seizure	DBN, cerebellar syndrome	HT, cholecystectomy	Unknown	Mg i.v.	y	y	Nystagmus, gait ataxia
(12)	32	m	Left side weakness, imbalance	Dysarthria, hypoesthesia, limb/gait ataxia, hemiparesis	Connective tissue disease	Gitelman syndrome	Mg + K p.o.	y	n	
(13)	74		Subacute slurred speech, impaired gait, irritability, diarrhea	Nystagmus, dysarthria, dysmetria, gait ataxia	HT, hyperlipidemia, seizure	PPI, diarrhea	Mg + Ca i.v. PPI stop	y	n	No
(14)	60	m	Thoracic discomfort, vertigo, nausea, ataxia	Ataxia, tremor, dysarthria, nystagmus, impaired walking	Alcohol abuse, HT, coronary artery disease, diarrhea	PPI	Mg, Ca p.o.	y	y	Could walk and eat
(15)	42	m	Anorexia, nausea, headache, dizziness			PPI	PPI stop	y	n	No
(16)	61	w	Weakness, gait impairment, cognitive impairment	Nystagmus, dysmetria, gait ataxia, kinetic tremor, confusion	Obesity, HT, T2DM, obstructive sleep apnea	TRPM6 mutation	Mg i.v.		y	
(17)	41	m	Subacute dysarthria, oscillopsia, paresthesia, limb tremor	Dysarthria, head tremor, ataxia, confusion	T2DM, HT, GERD, weight loss, diarrhea	PPI	Mg i.v.	y	y	No

*Ref, references; y, years; m, male; f, female; i.v., intravenously; p.o., orally; Mg, magnesium; Ca, calcium; K, potassium; PPI, proton pump inhibitor; RLS, restless legs syndrome; DBN, downbeat nystagmus; HT, hypertension; T2DM, type 2 diabetes mellitus; MGUS, monoclonal gammopathy; GERD, gastroesophageal reflux disease; PNP, polyneuropathy; y, yes; n, no*.

**Figure 1:**
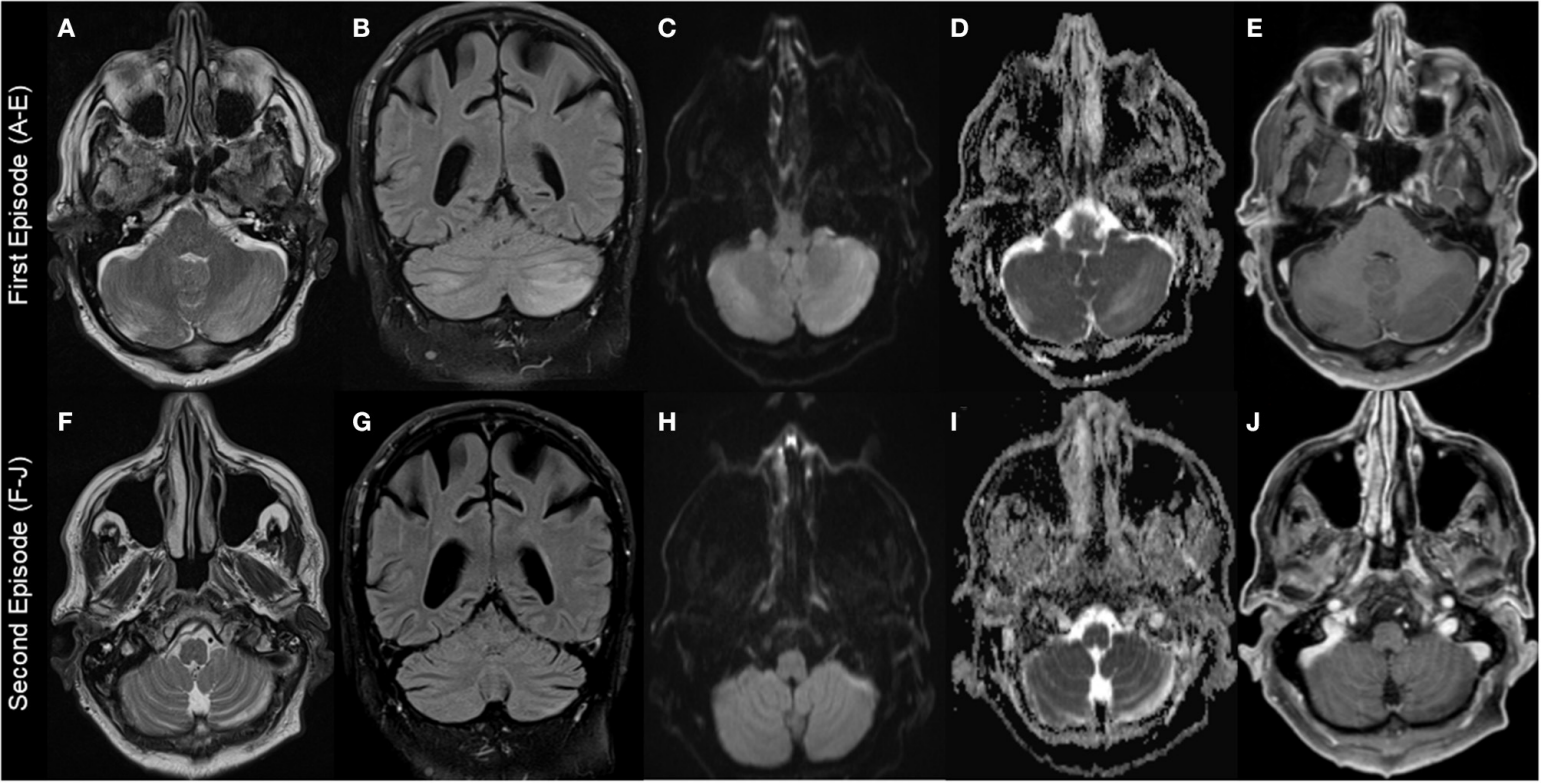
Brain MRI findings. First episode: Images **(A–E)**; second episodes **(F–J)** (resembling residual findings on further follow up MRIs): **(A+F)**: T2-weighted-images (T2W); **(B+G)**: fluid-attenuated inversion recovery (FLAIR) images; **(C+H)**: diffusion-weighted images (DWI); **(D+I)**: apparent diffusion coefficient (ADC) images; **(E+J)**: T1-weighted images (T1W) with gadolinium (Gd). Images show left-dominant cerebellar hyperintense lesions on T2W, FLAIR, and ADC images that are hypointense without Gd enhancement T1W images. DWI images at first episode showed hyperintense lesion and were normal at second episode.

Brain MRI was repeated revealing bilateral cerebellar edema that was interpreted as subacute stroke leading to a stroke work-up including transthoracic echocardiography, trans- and extracranial Doppler ultrasound and long-term ECG without pathological findings (Figure 1, Table 2). In addition, deficiency of folic acid and vitamin D and dyselectrolytemia (Table 3) were diagnosed, and calcium, folic acid, Mg (10 mmol per day), and vitamin D were supplemented orally. Importantly, Mg (10 mmol per day orally) was continuously taken from now on.

**Table 2:** Brain MRI features of reported cases.

**References**	**Brain MRI at admission**							**Follow-up brain MRI**
	**Location**	**Description (↑↓↔)**						
		**T2**	**FLAIR**	**DWI**	**ADC**	**T1**	**Gd**	
Case report	Bilateral cerebellar hemispheres	↑	↑	↑	↑	↓	–	Improved, but sequelae
(3)	Bilateral cerebellar hemispheres and vermis	↑						Improved, but sequelae
(4)	Cerebellar nodulus with swelling	↑		↑				Full resolution
(5)	Bilateral cerebellar hemispheres, vermis			↑				Improved, but sequelae
(5)	No acute abnormality							
(5)	Bilateral cerebellar hemispheres, cerebellar peduncles, effacement of sulci		↑	↔				
(6)	Left cerebellum		↑					Full resolution (4 weeks)
(7)	Normal							
(8)	Bilateral cerebellar hemispheres (symmetrical)	↑	↑	↑	↑		–	Full resolution, MR-spectroscopy: residual neuronal loss (3 months)
(9)	Bilateral cerebellar hemispheres without significant mass effect	↑	↑	↑	↑		–	Resolution of signal abnormality, volume loss, MR-spectroscopy: neuronal loss
(10)	Normal							
(11)	Cerebellar vermis (nodulus)		↑	↔			–	Full resolution
(12)	Bilateral cerebellar hemispheres, cortical atrophy	↑	↑					Improvement
(13)	Bilateral cerebellar hemispheres	↑	↑					Full resolution
(14)	Bilateral cerebellar hemispheres (6 months before admission), right cerebellar hemisphere on admission	↑	↑					
(15)	Bilateral cerebellar vasogenic edema							Significant improvement
(16)	Normal							
(17)	Bilateral anterior lobe of the cerebellum, vermis (minor alterations of mamillary bodies, periaqueductal area, medial thalami region)		↑	↑	↑			Full resolution

*Ref, References; MR, magnetic resonance; T2, T2-weighted images; FLAIR, fluid-attenuated inversion recovery; DWI, diffusion-weighted imaging; ADC, apparent diffusion coefficient; T1, T1-weighted images; Gd, gadolinium enhancement (+, yes; –, no); ↑, hyperintense; ↓, hypointense; ↔, isointense/normal*.

**Table 3:** Laboratory features of reported cases.

**References**	**Magnesium (NR: 0.7–1.05)**	**Potassium (NR: 3.5–4.5)**	**Phosphate (NR: 0.87–1.45)**	**Calcium (NR: 2.20–2.55)**
**Case report**				
1 episode	0.16	2.9	1.25	1.65
2 episode		2.9		2.0
3 episode	<0.1	3.2	0.83	1.76
(3)	0.19	2.4	0.59	Normal
(4)	<0.08			1.73
(5)	0.22	3.3	Normal	1.77
(5)	<0.2		1.27	2.24
(5)	<0.2	2.8	1.19	1.99
(6)	<0.1	Normal		Normal
(7)	0.1	2.8		
(8)	<0.28			1.85
(9)	0.15	Normal	Normal	1.59
(10)	<0.1–0.38	3.4		1.95
(11)	0.28	2.3	0.68	1.63
(12)	0.4	3.1	1.03	2.48
(13)	<0.12	3.2		1.25
(14)	0.16	Normal	Normal	1.7
(15)	0.16		1.23	1.63
(16)	1.5 mEq/L			
(17)	<0.21	2.45		1.83

*Values are in mmol/L if not otherwise stated; NR, normal range*.

Because of persistent symptoms, the patient was transferred to a neurorehabilitation ward. The diagnosis of stroke was questioned and cerebellitis was suspected. Therefore, a spinal MRI, extensive blood examinations with regard to paraneoplastic, autoimmune, and infectious diseases (anti-amphiphysin, anti-CV2, anti-PNMA2, anti-Ri, anti-Yo, anti-Hu, anti-recoverin, anti-SOX1, anti-Titin, anti-GAD, anti-NMDA, anti-VGCC, anti-LGi1, anti-CASPR2, GQ1b, anti-MAG, acetylcholine-receptor-antibodies, ANA, anti-dsDNA, anti-TPO, HIV, syphilis, lyme borreliosis, HHV6, herpes simplex, cytomegalovirus, vitamin B_12_, toxoplasmosis, herpes zoster, enterovirus, influenza) as well as an abdominal ultrasonography was performed and found to be normal. The cerebrospinal fluid (CSF) analysis were normal (including FACS analysis and cytology) except for an elevated protein of 0.7 g/L and mirrored oligoclonal bands (OCBs) attributed to the already known MGUS. There was a steady improvement of symptoms under continuous Mg, calcium, folic acid, and vitamin D supplementation, and after 5 months, the only patient's complaint was about rare movement-associated vertigo for a few seconds. The then performed neurological examination and brain MRI were normal.

### Second Episode 2017

In July 2017, the patient was hospitalized due to vertigo, nausea, vomiting, and inability to walk for the past 3 days. Medication was unchanged and especially oral Mg substitution (10 mmol daily) was continuously taken since 2015. The neurological examination showed saccadic eye movements, bilateral intention tremor of the arms, and severe gait ataxia.

Brain MRI revealed recurrent edema in the left cerebellar hemisphere. Recurrent cerebellitis was suspected and most examinations that were performed during the first episode in 2015 were repeated including the extensive blood examinations as described earlier and a CSF analysis, but reported to be normal despite the known elevated protein and mirrored OCBs in the CSF. In addition, FDG-PET and dermatological and urological examination for tumor screening were negative. Laboratory examination showed slight dyselectrolytemia that was orally substituted; Mg was not controlled (Table 3). Pulse therapy with glucocorticoids (1,000 mg methylprednisolone for 3 days) with oral tapering off was administered with clear clinical improvement supporting the diagnosis of cerebellitis. Neurorehabilitation was performed with further clinical improvement and eventually the patient only complained about residual vertigo when turning around and intermittent nausea. The neurological examination showed a pathologic vestibulo-ocular reflex suppression, mild bilateral ataxia in the finger-to-nose test (FNT), and impaired tandem walking. There was no relevant impact on activities of daily living. Medication was continued unchanged.

### Third Episode 2019

In September 2019, the patient was again hospitalized due to progressive walking difficulties leading to an inability to walk. Medication was again unchanged and especially oral Mg substitution (10 mmol daily) was continuously taken since 2017. Neurological examination showed first-degree nystagmus, bilateral intention tremor in the FNT, and severe gait ataxia.

Brain MRI showed unchanged hyperintensities in the left cerebellum. Recurrent cerebellitis was suspected and blood examinations with regard to paraneoplastic and infectious diseases (borreliosis, syphilis, anti-amphiphysin, anti-CV2, anti-PNMA2, anti-Ri, anti-Yo, anti-Hu, anti-recoverin, anti-SOX1, anti-Titin), a CSF analysis, and a spinal MRI were repeated and normal or without new information. In addition, a thoraco-abdominal CT, Aquaporin-4 antibodies, and anti-MOG antibodies were performed and normal. Glucocorticoid pulse therapy resulted in an improvement of limb ataxia with persisting gait ataxia. In the following month, including neurorehabilitative measures, there was an improvement of symptoms, and in February 2020, there was only residual vertigo during turning movements and intermittent nausea. Brain MRI was unchanged. Laboratory examinations showed persistent dyselectrolytemia including severe hypomagnesemia <0.1 mmol/l, severe hypocalcemia, and hypokalemia (Table 3) leading to the diagnosis of hypomagnesemia-induced cerebellar syndrome (HiCS).

In an endocrinological work-up, the patient denied regular consumption of alcohol or use of diuretics. He had no gastrointestinal symptoms (i.e., diarrhea or vomiting) and nutrition history was unremarkable as was his family history. Medications remained unchanged to 2015 (cf. previous section). Despite continuous oral Mg substitution since 2015 (10 mmol daily), blood analysis demonstrated persistent hypomagnesemia (0.4 mmol/L), but serum potassium and calcium had normalized. Blood gas analysis was normal, and calcium and magnesium excretion in the 24-h urine (24 h) were very low with a fractionated magnesium excretion rate of <1%. Based on these results, the diagnosis of extrarenal magnesium wasting was made and other differential diagnoses as Gitelman or Bartter syndrome were excluded. Proton pump inhibitor (PPI)–induced hypomagnesemia was considered as the most probable cause and the medication was stopped. In the following weeks, Mg normalized without improvement of clinical symptoms.

## Literature Review

A literature review with regard to hypomagnesemia-induced cerebellar syndromes was performed in PubMed, Google scholar, and Google using among others the following terms: “hypomagnesemia cerebellar,” “hypomagnesemia ataxia,” “low magnesium cerebellar,” “hypomagnesemia brain oedema,” “hypomagnesemia cerebral oedema,” “hypomagnesemia cerebellar oedema,” and “hypomagnesemia Posterior Reversible Leukoencephalopathy Syndrome.” We only included reports that referred to clinical, laboratory, and MR-imaging data for quality reasons. We identified 17 cases, so including our case, 18 cases contributed to this review. Table 1 summarizes the clinical, Table 2 the brain MRI, and Table 3 the laboratory features of all the cases.

## Discussion

HiCS seems to be a distinct disease entity due to the remarkable similarities of clinical, laboratory, and MR-tomographical features of the reported cases as summarized in Tables 1–3.

In the majority of cases, there was a subacute onset of predominately cerebellar symptoms that progressed over time leading to a delayed hospitalization of usually days to weeks. Symptoms included vertigo, nausea, vomiting, dysarthria, oscillopsia, and walking impairments up to the inability to walk. Additional symptoms were, among others, headache, cognitive impairment, somnolence, and confusion, and 7/18 patients had newly occurring hypomagnesemia-related seizures before or during hospitalization (Table 1).

The neurological examination on admission predominately yielded cerebellar symptoms including severe dysarthria, cerebellar ataxia, and nystagmus. Different forms of nystagmus were described; however, downbeat nystagmus as reported in 6/18 patients seems to be quite typical and a result of the frequent affection of the vermis and cerebellar nodulus as seen on MRI (Table 2) (4–7, 11). In addition, periodic/intermittent nystagmus might hint to HiCS as well being an overall rare neurological presentation (4, 7).

MRI findings were remarkably similar across the reports showing edema affecting the bilateral cerebellar hemispheres (11/18 patients) and/or the cerebellar vermis/nodulus (5/18 patients). Only in one case, unilateral edema of the cerebellar hemispheres was reported and 4/18 MRIs were normal despite severe clinical symptoms (Table 2). Noteworthy, only one case report described minor MR findings outside the cerebellum (17). MRI abnormalities were all hyperintense on T2-weighted images (T2W), fluid-attenuated inversion recovery (FLAIR) images, diffusion-weighted images (DWI), and apparent diffusion coefficient (ADC) images, and hypointense without gadolinium (Gd) enhancement on T1-weighted images (T1W). However, abnormalities were not always present in all sequences in the individual patients with T2 and ADC images possibly being most sensitive (Figure 1, Table 2).

Laboratory analysis showed severe hypomagnesemia almost always below 0.2 mmol/L, and frequently secondary potassium and calcium deficiency (Table 3).

Most common etiologies of hypomagnesemia were the usage of PPIs in 12 of 18 patients, alcohol abuse (4/18 patients) or intestinal malabsorption due to relevant gastrointestinal diseases/gastric surgery (3/18 patients). This might explain the predominately older population with a mean age of 58 years (±14.2 SD) and a range from 32 to 80 years.

In the vast majority of cases, the diagnosis of hypomagnesemia as was delayed by days to weeks, rarely even months to years. Initial false diagnosis included cerebellitis, paraneoplastic disorders, immune-mediated syndromes, meningitis/encephalitis, Wernicke encephalopathy, neurodegenerative process or toxic conditions leading to extensive redundant diagnostic work-ups including laboratory analysis with regard to paraneoplastic, infectious, and immune-mediated disorders, CSF analysis, tumor screening using imaging methods (CT, MRI, PET, sonography), urologic and dermatologic examinations, coloscopy and gastroscopy as well as stroke work-ups. Therefore, various redundant treatments were performed including intravenous antibiotics/acyclovir, steroid treatment, thiamine substitution, or stroke prophylaxis.

After the diagnosis of HiCS, Mg was mostly substituted intravenously leading to a rapid clinical and MR-tomographical improvement in all cases. However, approximately half of the patients had residual cerebellar symptoms affecting daily life. Correspondingly, MRI showed residual signs of atrophy and neuronal loss in approximately half of patients (Figure 1, Table 2). Furthermore, recurrent disease course was reported in 6/18 patients due to initial misdiagnosis or insufficient treatment (Table 1). Daily magnesium serum levels are highly variable depending on dietary intake, actual renal function/excretion, mobilization from bone/soft tissue, and endocrine factors (i.e., impact of endocrine regulators as PTH or calcitonin). Gastrointestinal magnesium wasting (i.e., caused by PPI treatment) usually does not cause gastrointestinal symptoms. Therefore, we hypothesize that our patient had a persistent severe magnesium deficiency due to ongoing PPI treatment which itself abolishes the effect of peroral repletion of magnesium stores. This could be an explanation for recurrent HiCS in our patient.

The delay in diagnosing HiCS and the recurrent disease course in some patients might have contributed to a high percentage of patients recovering with residual neurological deficits. Importantly, the delay in diagnosis and treatment led, among others, to seizures in 7/18 patients, arrythmia, cognitive impairment and somnolence, and in one patient, to secondary hydrocephalus due to cerebellar swelling with fourth ventricle compression requiring external ventricular drain insertion (5). These manifold complications further highlight the importance of properly diagnosing and treating hypomagnesemia as early as possible to avoid potentially life-threatening conditions. Immediate IV Mg substitution seems to have a quick impact on these conditions, and is therefore the most important therapeutic action. The positive effect of the glucocorticoid pulse therapy in our patient remains elusive because glucocorticoids do not lead to higher Mg levels. A positive effect on the cerebellar edema could however be postulated.

Because Mg is usually not part of the routine electrolyte panel, the probability of underdiagnosing hypomagnesemia-induced diseases is high, and physicians must be alert to hypomagnesemia-related diseases and the severity of its consequences (2). On the other hand, electrolyte changes must be taken seriously, and reduced potassium and/or calcium values could hint to severe hypomagnesemia (Table 3).

Depending on the pathophysiology, hypomagnesemia can be classified into three main groups: (1) decreased intake and/or redistribution, and (2) renal and (3) gastrointestinal losses. Redistribution from extra- to intercellular compartment occurs in refeeding syndrome or hungry bone syndrome after parathyroidectomy. Renal Mg wasting can result from various genetic defects leading to alterations in renal electrolyte handling (i.e., Bartter or Gitelman syndrome) or is acquired in the setting of medications (i.e., PPI, diuretics, cisplatin, aminoglycoside antibiotics, cyclosporine), alcohol abuse, or hypercalcemia. Hypomagnesemia due to gastrointestinal losses arises in patients with diarrhea, vomiting, malabsorption syndromes, or after bariatric surgery. PPIs rank among the most prescribed medications worldwide and therapy often lasts for months or even years. However, a clearly underestimated side effect of long-term treatment with PPI is promotion of gastrointestinal Mg wasting probably through a direct inhibitory effect on active intestinal Mg absorption mechanisms (18). This can lead to depletion of total body Mg stores causing severe hypomagnesemia in the complete absence of any gastrointestinal symptoms (19–21). Diagnosis of the etiology of hypomagnesemia usually is straight forward. Detailed assessment of the patient's history focuses on dietary habits, daily alcohol consumption, actual or past (i.e., st. p. chemotherapy with platin-based substances) medications and gastrointestinal symptoms. Laboratory evaluation includes, apart from measurement of serum Mg and potassium, assessment of calcium homeostasis (corrected serum calcium and PTH) and blood gases in cases with suspected genetic causes of renal magnesium loss (Bartter or Gitelman syndrome). Remarkably, serum Mg represents <1% of total body Mg and therefore correlates poorly with Mg stores and intracellular Mg content (22). The most useful diagnostic step allowing discrimination between renal and gastrointestinal Mg losses is measurement of renal Mg excretion in the 24 h urine. Daily excretion of more than 10 to 30 mg or a fractional excretion of Mg above 2% is an indicator of renal Mg wasting (23). Treatment of hypomagnesemia involves cessation of triggers (i.e., stopping/reducing medications) and supplementation of magnesium by oral or parenteral route. Route of administration and aggressiveness of correction depends on severity of the clinical symptoms and signs.

In our opinion, HiCS seems to be a distinct disease entity that can be differentiated from posterior reversible encephalopathy syndrome (PRES). In PRES, elevated blood pressure plays a critical role in the majority of patients. In addition, PRES is frequently observed in patients with (pre)eclampsia, sepsis, renal diseases, or during treatment regimens with immunosuppressive or cytotoxic agents (1). These conditions did not apply to the majority of our patients with hypertension only reported in 2/18 patients on admission that was quickly reversible under Mg substitution (24). PRES often presents with quantitative and qualitative disorders of consciousness, headache, visual disturbances (hemianopia, visual neglect, visual hallucinations, cortical blindness) which were not the leading symptoms in our case series except for seizures in 7/18 patients that are reported in PRES in >70% of patients (24). Typical MRI features in PRES are bilateral, frequently symmetric distributed lesions that follow a parieto-occipital pattern in about 70%. In addition, frontal and temporal lobe involvement and subcortical white matter lesions are common (24, 25). Lesions in other areas such as the cerebellum, brain stem, basal ganglia, or the spinal cord are less common and show patchy and rounded foci (26, 27). In contrast, lesion in our case series were exclusively limited to the cerebellum showing a laminar and homogeneous pattern except for one case reporting minor alterations outsight the cerebellum that might be a result of concomitant diseases (17). MR lesions of PRES and HiCS share the same pattern being hyperintense in T2W, FLAIR, DWI, and ADC images and iso- or hypointense without Gd enhancement on T1W imaging resembling edema. In contrast, Gd-enhancing lesions are described in about 20% of PRES. We cannot elute to microhemorrhages that are described in up to 65% in PRES because it was not reported in our case series (28). With regard to pathophysiology, PRES and HiCS might share similar mechanisms. In both diseases, endothelial dysfunction with capillary leakage, blood–brain barrier disruption, and axonal swelling leading to cerebral edema are often postulated. However, the cause seems different predominantly being hypomagnesemia in HiCS and hypertension in PRES (6, 24).

Differentiation to acute cerebellitis can be challenging because of similar clinical presentations with subacute cerebellar symptoms and similar pattern on MRI with cerebellar hyperintensities in T2, FLAIR, DWI, and ADC sequences in the majority of cases. However, in one case series, fever and headache were present in 88 and 71% of patients, Gd enhancement (cortical and leptomeningeal) in 78% of patients, median leukocyte count in the CSF was 104 (range 0–797), and an etiology (infectious, paraneoplastic, medication induced) was found in 66% of cerebellitis cases (29). These differences can help in differentiating cerebellitis from HiCS.

Paraneoplastic cerebellar degeneration (PCD) can be distinguished from HiCS by a slowly progressing non-relapsing clinical course with cerebellar symptoms over weeks to months. Additional neurologic presentations such as encephalomyelitis or Lambert–Eaton syndrome may be present depending on the cause of the disease (30). Identifying the underlying neoplasm such as lung cancer, gynecologic cancer, breast cancer, and lymphoma including testing for paraneoplastic antibodies such as anti-Yo, anti-Tr, or antimetobotropic glutamate receptor 1 (mGluR1) is crucial in the diagnosis of PCD (30). MRI is mostly normal helping to rule out differential diagnosis. Rarely, there is contrast enhancement of the cerebellar folia, and in the course of the disease, there is a cerebellar atrophy (31). CSF analysis may reveal mild pleocytosis and a mildly elevated protein, which despite the MRI, may help to distinguish PCD from HiCS.

Overall, clinicians should be suspicious with regard to HiCS in patients with subacute onset of predominately cerebellar symptoms as well as seizures of unknown origin in patients taking PPIs, having relevant gastrointestinal diseases/gastric surgery or alcohol abuse. In the clinical examination, downbeat and periodic/intermittent nystagmus seems to be quite typical for HiCS. Mg concentration in the blood and brain MRI helps to confirm the diagnosis in most cases, the latter showing cerebellar edema as described earlier and in Table 2 and Figure 1. In addition, differential diagnosis such as PRES and cerebellitis as well as paraneoplastic, infectious, or autoimmune diseases should be ruled out (24–31).

## Conclusion

HiCS seems to be a distinct disease entity because of the remarkable similarities of clinical, laboratory, and MR-tomographical features. It should be diagnosed and treated early to avoid recurrent disease courses, residual symptoms, and potentially life-threatening conditions such as seizures. Physicians must be alert to hypomagnesemia-induced symptoms including HiCS as Mg is usually not part of the routine electrolyte panel.

## Data Availability Statement

The original contributions presented in the study are included in the article/supplementary material, further inquiries can be directed to the corresponding author/s.

## Ethics Statement

Written informed consent was obtained from the individual(s) for the publication of any potentially identifiable images or data included in this article.

## Author Contributions

All authors listed have made a substantial, direct and intellectual contribution to the work, and approved it for publication.

## Conflict of Interest

The authors declare that the research was conducted in the absence of any commercial or financial relationships that could be construed as a potential conflict of interest.
